# Supplementary material for: An Engineered Receptor-Binding Domain Improves the Immunogenicity of Multivalent SARS-CoV-2 Vaccines
Source: mBio. 2021 May 11;12(3):e00930-21. doi: 10.1128/mBio.00930-21 (PMC8262850; doi:10.1128/mBio.00930-21)
Supplement: FIG S1 [file mbio.00930-21-sf001.pdf]

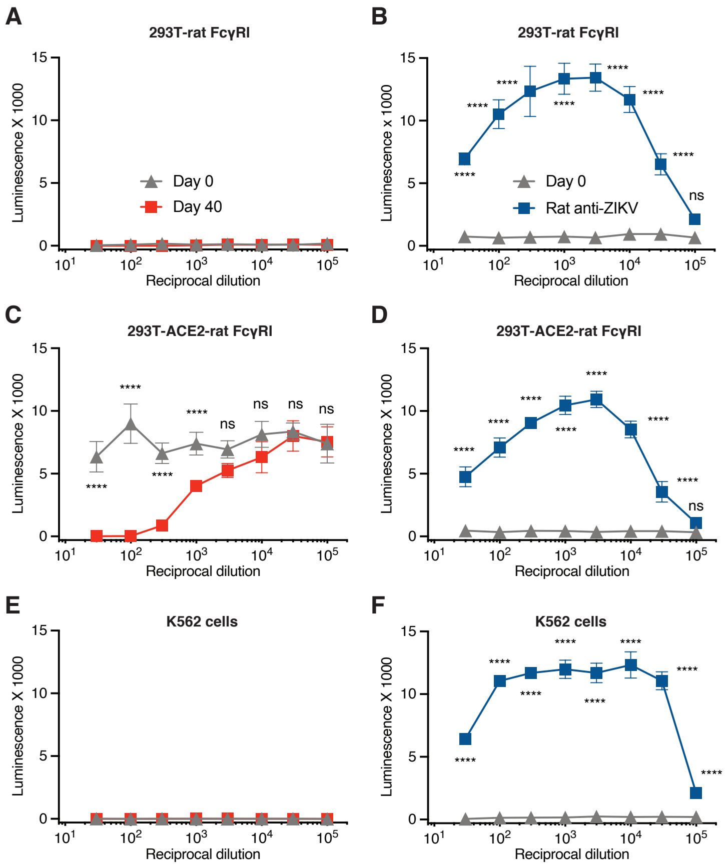


**Fig. S1. Anti-RBD antisera does not mediate antibody-dependent enhancement of SARSCoV-2 S protein-mediated entry.** (**A**) HEK293T cells were transfected to express rat FcγRI and infected with SARS2-PV in the presence of the indicated dilutions of preimmune day 0 or immune day 40 antisera. (**B**) Experiments similar to those in panel A except that ZIKV VLP and anti-ZIKV antisera, also elicited in Sprague Dawley rats, were compared to preimmune sera. (**C**, **D**) Experiments identical to those in panel A and B respectively except that HEK239T cells stably expressing human ACE2 were again transfected to express rat FcγRI before incubation with SARS2-PV (**C**) or ZIKV-VLP (**D**). (**E**, **F**) Experiments identical to A and B, respectively, except that the pro-monocytic K562 cell line was incubated with SARS2-PV (E) or ZIKV-VLP (F). Error bars in panels A-F indicate the range of two experiments run in parallel. Significant differences with day-0 preimmune sera are indicated (**** indicates $P<0.001$ by two-way ANOVA; ns indicates not significant).
