# Supplementary material for: An Engineered Receptor-Binding Domain Improves the Immunogenicity of Multivalent SARS-CoV-2 Vaccines
Source: mBio. 2021 May 11;12(3):e00930-21. doi: 10.1128/mBio.00930-21 (PMC8262850; doi:10.1128/mBio.00930-21)
Supplement: FIG S2 [file mbio.00930-21-sf002.pdf]

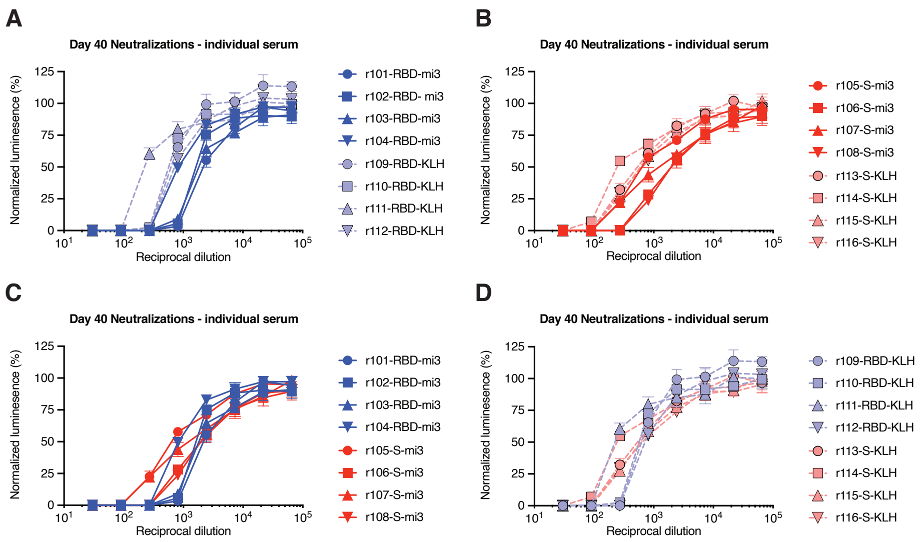

Fig. S2. RBD/Spike mi3/KLH neutralizations for individual rats. Four female Sprague Dawley rats for each group were inoculated with either RBD-Spytag or S-protein-Spytag conguated to either Spycatcher-mi3 particles by isopeptide bond formation, or KLH by EDC. Sera were harvested at day 45 and each serum was compared for its ability to neutralize S-protein-pseudotyped retroviruses (SARS2-PV), by measuring the activity of a firefly-luciferease reporter expressed by these pseudoviruses. (**A**) RBD conjugated to mi3 vs. KLH. (**B**) S-protein conjugated to mi3 vs. KLH. (**C**) mi3 conjugates of RBD vs S-protein. (**D**) KLH conjugates of RBD vs S-protein.
