# Supplementary material for: An Engineered Receptor-Binding Domain Improves the Immunogenicity of Multivalent SARS-CoV-2 Vaccines
Source: mBio. 2021 May 11;12(3):e00930-21. doi: 10.1128/mBio.00930-21 (PMC8262850; doi:10.1128/mBio.00930-21)
Supplement: FIG S3 [file mbio.00930-21-sf003.pdf]

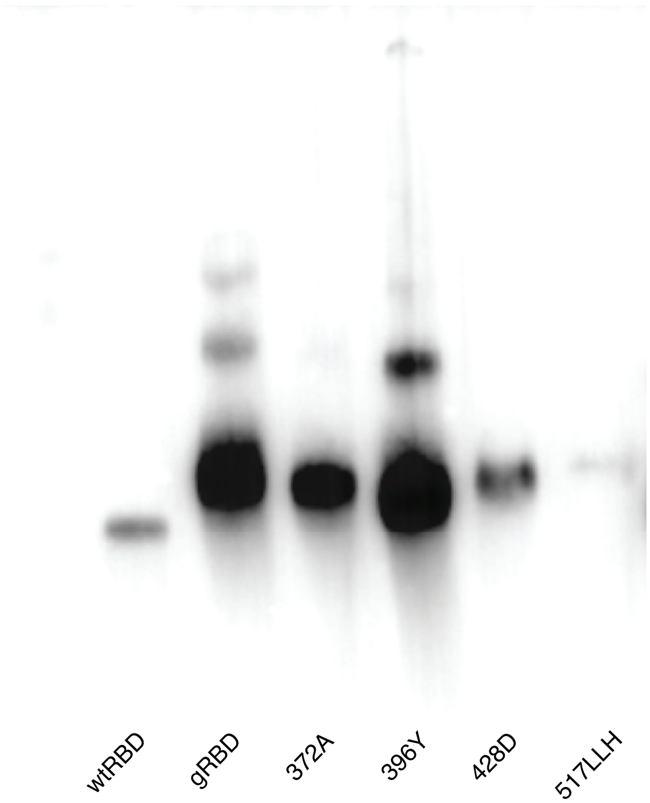

370	-	+	-	+	+	+
394	-	+	+	-	+	+
428	-	+	+	+	-	+
517	-	+	+	+	+	-

Fig. S3. Individual glycosylation motifs promote expression of a multimeric RBD fusion protein. SARS-CoV-2 RBD variants with different combinations of glycosylation motifs, as indicated, were expressed as fusions to the C-terminus of *H. pylori* NAP 12-mer. Native western blots probed with ACE2-Fc-HRP were performed on Expi293 supernatants harvested 5 days post transfection.
