# Supplementary material for: An Engineered Receptor-Binding Domain Improves the Immunogenicity of Multivalent SARS-CoV-2 Vaccines
Source: mBio. 2021 May 11;12(3):e00930-21. doi: 10.1128/mBio.00930-21 (PMC8262850; doi:10.1128/mBio.00930-21)
Supplement: FIG S4 [file mbio.00930-21-sf004.pdf]

A

```
gRBD-Fc
MKHLWFFLLLVAAPRWVLSNITNLCPFGEVFNATRFASVYAWNRKRISNCVADYSVLYNSTSFSTFKCYGVSPTKLN
DLCFTNVTADSFVIRGDEVRQIAPGQTGKIADYNYKLPDNFTGCVIAWNSNNLDSKVGGNYNYLYRLFRKSNLKPFE
RDISTEIYQAGSTPCNGVEGFNCYFPLQSYGFQPTNGVGYQPYRVVVLSFENLTAPATVCGPGGSGGSDKTHTCPPC
PAPELLGGPSVFLFPPKPKDTLMISRTPEVTCVVVDVSHEDPEVKFNWYVDGVEVHNAKTKPREEQYNSTYRVVSVL
TVLHQDWLNGKEYKCKVSNKALPAPIEKTISKAKGQPREPQVYTLPPSRDELTKNQVSLTCLVKGFYPSDIAVEWES
NGQPENNYKTTPPVLDSDGSFFLYSKLTVDKSRWQQGNVFSCSVMHEALHNHYTQKSLSLSPGK

gRBD-foldon
MKHLWFFLLLVAAPRWVLSNITNLCPFGEVFNATRFASVYAWNRKRISNCVADYSVLYNSTSFSTFKCYGVSPTKLN
DLCFTNVTADSFVIRGDEVRQIAPGQTGKIADYNYKLPDNFTGCVIAWNSNNLDSKVGGNYNYLYRLFRKSNLKPFE
RDISTEIYQAGSTPCNGVEGFNCYFPLQSYGFQPTNGVGYQPYRVVVLSFENLTAPATVCGPGGSGGGGSGPGGYIP
EAPRDGQAYVRKDGEWVLLSTFLGSGGGEPEA

NAP-gRBD
MKHLWFFLLLVAAPRWVLSMKTFEILKHLQADAIVLFMKVHNFHWNVKGTDFFNVHKATEEIYEGFADMFDDLAERI
AQLGHHPLVTLSEALKLTRVKEETKTSFHSKDIFKEILEDYKHLEKEFKELSNTAEKEGDKVTVTYADDQLAKLQKS
IWMLQAHLAGGGSGGGPGSGNITNLCPFGEVFNATRFASVYAWNRKRISNCVADYSVLYNSTSFSTFKCYGVSPTKL
NDLCFTNVTADSFVIRGDEVRQIAPGQTGKIADYNYKLPDNFTGCVIAWNSNNLDSKVGGNYNYLYRLFRKSNLKPF
ERDISTEIYQAGSTPCNGVEGFNCYFPLQSYGFQPTNGVGYQPYRVVVLSFENLTAPATVCGPGSGGGEPEA

gRBD-ferritin
MKHLWFFLLLVAAPRWVLSDYKDDDDKGGGPNITNLCPFGEVFNATRFASVYAWNRKRISNCVADYSVLYNSTSFST
FKCYGVSPTKLNDLCFTNVTADSFVIRGDEVRQIAPGQTGKIADYNYKLPDNFTGCVIAWNSNNLDSKVGGNYNYLY
RLFRKSNLKPFERDISTEIYQAGSTPCNGVEGFNCYFPLQSYGFQPTNGVGYQPYRVVVLSFENLTAPATVCGPGGG
SGGTGGDIIKLLNEQVNKEMNSANLYMSMSSWAYTHSLDGAGLFLFDHAAEEYEHAKKLIIFLNENNVPVQLTSISA
PEHKFEGLTQIFQKAYEHEQHISESINNIVDHAIKSKDHATFNFLQWYVAEQHEEEVLFKDILDKIELIGNENHGLY
LADQYVKGIAKSRKS

gRBD-mi3
MKHLWFFLLLVAAPRWVLSNITNLCPFGEVFNATRFASVYAWNRKRISNCVADYSVLYNSTSFSTFKCYGVSPTKLN
DLCFTNVTADSFVIRGDEVRQIAPGQTGKIADYNYKLPDNFTGCVIAWNSNNLDSKVGGNYNYLYRLFRKSNLKPFE
RDISTEIYQAGSTPCNGVEGFNCYFPLQSYGFQPTNGVGYQPYRVVVLSFENLTAPATVCGPGSSGSSGGSGGSMKM
EELFKKHKIVAVLRANSVEEAKKKALAVFLGGVHLIEITFTVPDADTVIKELSFLKEMGAIIGAGTVTSVEQARKAV
ESGAEFIVSPHLDEEISQFAKEKGVFYMPGVMTPTELVKAMKLGHTILKLFPGEVVGPQFVKAMKGPFPNVKFVPTG
GVNLDNVCEWFKAGVLAVGVGSALVKGTPVEVAEKAKAFVEKIRGCTEGGSGKLGSIEFIKVNKGSGEPEA
```

B

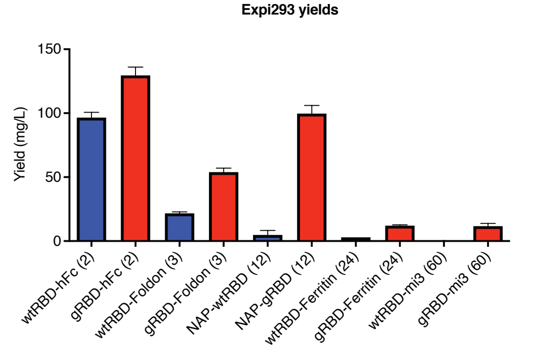

C

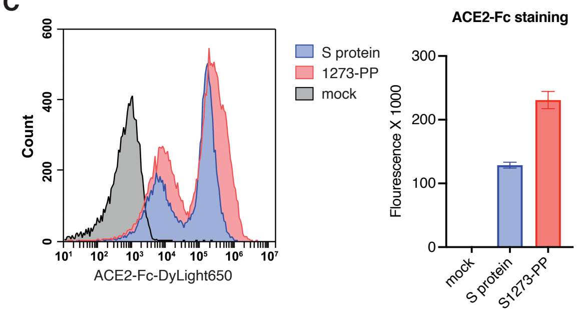

Fig. S4. Multivalent gRBD fusion proteins express more efficiently than their wtRBD counterparts. (**A**) Complete sequences of the multivalent gRBD fusion constructs used in these studies. Green indicated signal peptide; black, linker residues; blue, multivalent carrier protein; red, gRBD; purple, affinity tag. (**B**) Yields of purified wtRBD and gRBD multimers expressed from the CMV/R vector in Expi293 cells. Values reflect a minimum of two independent transfections. Error bars indicate s.d. (**C**) Flow cytometery of 293T cells transiently transfected with SARS-CoV2 S protein in the pCAGGS vector, or S1273-PP in the CMVR vector. Expression was measured by by staining with ACE2-Fc-DyLight650. Right panel indicates mean fluorescence intensity as determine from the histogram shown in the left panel.
